# Association Between the 2023 Kahramanmaraş Double Earthquake and Pathogen Distribution in Periprosthetic Joint Infection After Knee Arthroplasty

**DOI:** 10.3390/jcm15114006

**Published:** 2026-05-22

**Authors:** Osman Çiloğlu, Evren Karaali, Hakan Uslu, Oğuzhan Çiçek, Mehmet Yiğit Gökmen, Özhan Pazarcı, Mustafa Çıtak

**Affiliations:** 1 Department of Orthopedics and Traumatology, University of Health Sciences Adana City Training and Research Hospital, 01250 Adana, Turkey; drevrenkaraali@gmail.com (E.K.); hakanusl77@gmail.com (H.U.); oguzhan.cicek21@hotmail.com (O.Ç.); dr.pazarci@gmail.com (Ö.P.); 2 Department of Orthopedics and Traumatology, Çanakkale Onsekiz Mart University, 17020 Canakkale, Turkey; mehmet_yigit_gokmen@hotmail.com; 3 Department of Orthopaedic Surgery, Knappschaft Kliniken Dortmund, 44137 Dortmund, Germany; mcitak@gmx.de

**Keywords:** periprosthetic joint infection, total knee arthroplasty, earthquake, polymicrobial infection, level of evidence: Level III, retrospective comparative study

## Abstract

**Background:** Periprosthetic joint infections (PJIs), a significant complication of total knee replacement surgery, are influenced by patient, surgeon, and healthcare system factors. Natural disasters can disrupt healthcare services and alter microbiological factors in the hospital environment. The impact of natural disasters on pathogen distribution in periprosthetic joint infection (PJI) is unclear. Therefore, this study investigated the association between the 2023 Kahramanmaraş-centered earthquakes in Türkiye and changes in microbiological patterns of PJI after knee arthroplasty. **Methods:** This retrospective cohort study included patients who developed PJI following total knee arthroplasty at the study center. The patients were divided into two groups based on the timing of their PJI diagnosis: pre-earthquake and post-earthquake. The demographic characteristics, comorbid diseases, and perioperative characteristics of each patient were recorded, and their microbiological profiles were analyzed. Logistic regression analysis examined the relationships between patient-related factors and causative agents. **Results:** 56 patients were studied and divided into two groups: 26 patients in the pre-earthquake group and 30 in the post-earthquake group. Furthermore, 79 bacterial isolates were obtained from these patients. Demographic, metabolic, and preoperative characteristics were similar between the two groups. No significant difference was found in the overall distribution of bacterial isolates. However, Gram-negative organisms, primarily Acinetobacter baumannii and Pseudomonas aeruginosa, increased in the isolate distribution after the earthquake. Patient analysis revealed that polymicrobial PJIs were significantly more frequent after the earthquake (56.7% vs. 23.1%; *p* = 0.011). Diabetes mellitus (DM) and smoking were associated with an increased risk of polymicrobial infection; the association was not statistically significant. **Conclusions:** In the post-earthquake period, patients who had undergone total knee arthroplasty and developed PJI showed a higher proportion of polymicrobial infections and a numerical increase in Gram-negative pathogens, along with more complex infection patterns compared to the pre-earthquake period. Although both patient groups demonstrated similar characteristics regarding patient-related and surgical factors, the observed changes indicate that the pressure on the healthcare system after a natural disaster can affect a hospital’s microbiological ecology. Identifying these indirect effects is crucial for guiding microbiological surveillance and infection control during post-disaster recovery periods, even for elective patients.

## 1. Introduction

Periprosthetic joint infections (PJIs) are one of the most severe complications after knee replacement surgery. PJIs lead to significant morbidity, prolonged hospital stays, repeated surgeries, and increased healthcare costs. Despite advanced surgical techniques, improved implant designs, and enhanced precautions to decrease the risk of preoperative infection, the incidence of periprosthetic joint infection (PJI) remains significant. This indicates that the development of PJI depends on several factors. The development of PJI is influenced by patient-related factors, surgical factors, microbiological characteristics, and healthcare system conditions.

Although large-scale natural disasters can increase the risk of infection and alter microbiological ecology, these relationships are poorly defined. The double earthquake that struck Türkiye on 6 February 2023 was centered around Kahramanmaraş and caused widespread destruction, mass displacement, and serious healthcare disruptions in 11 provinces [1,2,3]. Significantly increased patient load, hospital overcrowding, and deteriorating infection control measures were critical factors increasing infection risks after the earthquake [2,3,4].

Studies conducted on post-earthquake orthopedic patients have focused on trauma-related injuries such as open fractures, crush injuries, and those requiring fasciotomy or amputation. These studies have shown an increased frequency of Gram-negative and polymicrobial pathogens in infections observed in this patient group [5,6,7,8]. However, the impact of the earthquake on nontraumatic orthopedic patients, particularly those undergoing elective procedures such as total knee arthroplasty, has not been sufficiently addressed.

The effects of healthcare service disruptions following earthquakes extend beyond traumatic patients; they also alter musculoskeletal infection patterns in nontraumatic orthopedic patients. After 6 February 2023, following the Kahramanmaraş earthquake, an increase was reported in intensive care unit-acquired and Gram-negative bacterial infections among hospitalized adult and pediatric patients, including those not directly related to trauma complications [9,10,11]. These findings indicate that the pressure on healthcare services after an earthquake can widely alter the microbiological ecology, potentially impacting both traumatic and nontraumatic patients.

The microbiological agents and pathological processes of PJIs differ from those seen in musculoskeletal infections in traumatic patients. Intraoperative conditions and hospital bacterial flora are crucial to the development of PJI. Disruptions in infection control, such as limited hospital bed capacity and operating room logistics, can lead to changes in pathogen distribution in PJI, independent of surgical techniques and patient-related factors [12,13,14]. Several specific mechanisms may explain how earthquakes alter the hospital microbiological ecology: damage to water and sanitation infrastructure facilitates environmental contamination with Gram-negative organisms; massive influx of dust and soil during and after seismic events introduces environmental pathogens into clinical areas; sudden patient surges overwhelm standard isolation and decontamination protocols; and cross-contamination risk increases as healthcare workers manage exceptionally high patient volumes under resource-constrained conditions.

This study aimed to determine whether these effects observed in traumatic orthopedic patients are also reflected in the microbiological flora and infection patterns of primary total knee arthroplasty (PJI) patients, a nontraumatic group. We hypothesized that following the earthquake, healthcare system-related disruptions resulted in changes in the hospital microbiological environment, with an increased prevalence of Gram-negative pathogens and polymicrobial infections in patients developing PJIs.

## 2. Methods

### 2.1. Study Design and Patient Selection

This retrospective cohort study was approved by the local ethics committee and conducted at a tertiary referral center with substantial post-disaster patient volume following the 6 February 2023 earthquake. The pre-earthquake study period was defined as January 2020 through January 2023, during which 2653 primary total knee arthroplasties were performed at the study center. The post-earthquake study period was defined as July 2023 through March 2025; July 2023 was chosen as the start of this period as it represents the point at which institutional patient flows and healthcare delivery had begun to normalize following the acute post-disaster phase. During this period, 1764 primary total knee arthroplasties were performed. Only patients with a minimum follow-up of 12 months after the index arthroplasty were included, ensuring that late-onset PJI cases would not be missed due to insufficient observation time. Patients who developed PJIs during follow-up in either period and had complete microbiological documentation were included. Furthermore, patients were divided into two groups based on when PJI was confirmed by microbiological culture: the pre-earthquake group comprised patients with culture-confirmed PJIs diagnosed between January 2020 and January 2023 and the post-earthquake group comprised those diagnosed between July 2023 and March 2025. Culture-negative cases were excluded from the microbiological analysis; all 56 patients included in the study had at least one positive culture result, and no culture-negative cases were included in the overall distribution analysis. Patients with complete demographic, clinical, surgical, and microbiological data were included. Conversely, patients were excluded if they had prior surgery on the same knee or a diagnosis of rheumatological or peripheral artery disease that could increase the risk of infection. To ensure patient standardization, age, sex, body mass index (BMI), smoking status, and diabetes mellitus (DM) prevalence were evaluated and found to be comparable between the two groups. Although this was a single-center study, the study setting managed a high patient volume from a broad catchment area, particularly in the post-earthquake period. It should be acknowledged that other earthquake-related changes in healthcare delivery—including ward overcrowding, temporary modifications to operating room protocols, potential changes in antibiotic prophylaxis practices, staffing shortages, and disruptions to infection control infrastructure—could not be systematically quantified in this retrospective analysis and may have independently contributed to the observed shifts in pathogen distribution.

### 2.2. Surgical Procedure and Perioperative Management

All patients underwent surgery performed by the same surgical team using a medial parapatellar incision and the same knee prosthesis brand (ANTHEM Total Knee System, Smith & Nephew, Memphis, TN, USA). All patients received antibiotic prophylaxis (including cefazolin) in the preoperative period and underwent tourniquet application during surgery. Bleeding was controlled in all patients, systemic tranexamic acid was administered, and a hemovac drain was used. Routine preoperative assessments included blood parameters, such as anemia and serum albumin levels, that could predispose patients to infection or delayed wound healing, and patients with abnormal values did not undergo surgery. All patients received the same postoperative care and follow-up. The study ensured that differences in infection microbiology were minimally influenced by perioperative or postoperative factors.

### 2.3. Microbiological Assessment and Outcome Definition

Microbiological findings were interpreted according to the timing of the PJI diagnosis. Three periprosthetic tissue samples were obtained from all patients who developed PJIs during surgery. All samples were cultured using both aerobic and anaerobic techniques according to hospital protocols. Growth in two or more samples was considered indicative of infection; growth of the causative agent in a single sample was evaluated considering clinical factors and deemed potential contamination. A PJI was classified as an early infection if it developed within the first 3 months after primary surgery, a delayed infection if it developed between 3 and 12 months and a late infection if it occurred after 12 months. The presence of polymicrobial infection was determined as the primary outcome and analyzed as the dependent variable in the logistic regression analysis. PJI was diagnosed according to the diagnostic criteria determined at the 2018 International Consensus Meeting on Musculoskeletal Infections, based on clinical, microbiological, and laboratory findings.

### 2.4. Statistical Analysis

Continuous variables were expressed as the mean ± standard deviation or median (interquartile range), and categorical data were expressed as numbers (*n*) and percentages (%). The Shapiro–Wilk test assessed the normality assumption. Student’s t-tests were used to compare continuous variables and Chi-square or Fisher’s exact tests were used for categorical variables. Statistical significance was set at *p* < 0.05. Analyses were performed using IBM SPSS Statistics 26.0 (IBM Corp., Armonk, NY, USA). Regression analyses were considered exploratory due to the limited sample size.

## 3. Results

The study included 56 patients who developed PJIs following primary total knee arthroplasty. During the pre-earthquake period (January 2020–January 2023), 2653 primary total knee arthroplasties were performed, of which 26 patients developed PJI (PJI rate: 0.98%). During the post-earthquake period (July 2023–March 2025), 1764 primary total knee arthroplasties were performed, of which 30 patients developed PJIs (PJI rate: 1.70%). All patients had a minimum follow-up of 12 months after the index arthroplasty. These patients were divided into two groups based on when the PJI was diagnosed: pre-earthquake (*n* = 26) or post-earthquake (*n* = 30). Age, BMI, sex, smoking status, and DM prevalence were comparable between the two groups (*p* > 0.05). Moreover, surgical duration and preoperative laboratory parameters, including preoperative hematocrit and serum albumin levels, were similar in both groups (*p* > 0.05) (Table 1).

In total, 79 bacterial isolates were detected: 32 were pre-earthquake and 47 were post-earthquake. The overall distribution of infectious agents did not significantly differ between the pre- and post-earthquake periods (*p* = 0.141). A numerical increase in Gram-negative pathogens was observed in the post-earthquake period, although this did not reach statistical significance in the overall distribution analysis. The prevalence of Acinetobacter baumannii and Pseudomonas aeruginosa as causative agents increased post-earthquake. In contrast, a partial decrease was noted in the prevalence of Gram-positive pathogens, including methicillin-resistant Staphylococcus aureus and coagulase-negative staphylococci. Infection patterns significantly differed between the pre- and post-earthquake periods. Infections involving polymicrobial pathogens significantly increased in the post-earthquake period compared to the pre-earthquake period (56.7% vs. 23.1%; *p* = 0.011) (Table 2).

Logistic regression analysis revealed the relationship between patient-related factors and the occurrence of polymicrobial pathogens in PJIs (Table 3). Polymicrobial infections occurred more frequently in patients with DM and smokers. This analysis indicates that patients with type DM have a 2.05-fold increased risk of developing polymicrobial infections. Smokers had a 2.56 times higher rate of polymicrobial infections than nonsmokers. No significant difference was found between smoking and DM regarding the development of polymicrobial infections (DM: *p* = 0.257; smoking: *p* = 0.288). No significant difference was noted between BMI and the development of polymicrobial infection. Although not statistically significant, the direction and magnitude of the odds ratios indicate an increased possibility of polymicrobial infection in cases involving unfavorable living conditions and metabolic comorbidities.

In patients with post-earthquake polymicrobial PJIs (*n* = 17), clinical severity significantly worsened during follow-up. Seven patients (41.2%) were admitted to the intensive care unit at least once during infection management and 2 patients (11.8%) required two or more revision surgeries. Multidrug-resistant Gram-negative pathogens were detected in 2 patients (11.8%). These findings indicate that polymicrobial infections in post-earthquake PJIs are more complex and associated with increased resource utilization (Table 3).

## 4. Discussion

The study found that natural disasters increase the burden on healthcare services and can increase the complexity of musculoskeletal infections in both traumatic and nontraumatic orthopedic patients by altering microbiological environments. Compared to the pre-earthquake group, polymicrobial infections were more frequent, Gram-negative pathogens were more prevalent, and more complex infection patterns were observed in the post-earthquake patient group. These differences were observed despite similar patient demographics and clinical profiles, indicating that earthquake-related factors may be influential. In PJIs, grouping by the timing of microbiological diagnosis revealed how post-earthquake changes affected healthcare services, including the resulting healthcare burden and potentially changing microbiological flora during this period.

Consistent with previous studies, Gram-negative pathogens, particularly Acinetobacter baumannii and Pseudomonas aeruginosa, increased in frequency in the patient cohort after the earthquake [8,9,10]. This group of pathogens is known for their environmental persistence, resistance potential, and transmission in healthcare settings, particularly in cases involving inadequate infection control [9,10]. As previously shown, Gram-negative pathogens dominated infections in adult and pediatric patients hospitalized after 6 February 2023, following the Kahramanmaraş earthquake [11,12,13,14]. This supports the idea that increased patient volumes during these periods affect the microbiological flora of hospital environments. Moreover, increasing Gram-negative pathogen levels indicate that infection processes in healthcare facilities affected by earthquakes are more complex.

A significant finding of this study is the increase in polymicrobial PJIs (post-earthquake illnesses) [15]. The presence of polymicrobial agents indicates increased infection severity and is associated with treatment difficulties, higher surgical failure rates, longer hospital stays, and prolonged antibiotic use. Previous studies on earthquakes have shown increases in polymicrobial wound and soft tissue infections, showing increased burden on healthcare services, environmental contamination, delayed patient intervention, repeated surgeries, and cross-contamination [8,12,13]. Unlike previous studies, this study focused not only on traumatic patients but also on nontraumatic orthopedic patients after the earthquake. Furthermore, we demonstrated that changes in healthcare during this period can manifest in pathogens, changes in infection behavior, and increased severity.

Although logistic regression showed no association between type DM and smoking with polymicrobial pathogens, both demonstrated a clinically significant association with more than two probabilities. Metabolic dysregulation, an impaired immune response, microvascular damage in patients with DM, and low tissue oxygenation in smokers, along with an impaired immune system, predispose them to more complex infections. Parallel relationships between comorbid disease burden and infection severity have been found in post-earthquake patients with prolonged intensive care needs and increased hospital stays [14,16,17]. Our study analysis reveals that the lack of significant differences between the factors is due to the limited sample size. Rather than an increase in infection incidence, polymicrobial pathogens, identified as causative agents in post-earthquake infections, are associated with prolonged intensive care unit stays, repeated surgical failures, and multidrug-resistant Gram-negative organisms, indicating a high level of clinical severity. These situations show that changes in the healthcare system following natural disasters can affect the distribution of pathogens, clinical behavior, and the treatment difficulty of infections in traumatic and nontraumatic orthopedic patients.

Additionally, our study findings suggest that large-scale disasters, such as earthquakes, affect not only emergency trauma patients but also nontraumatic patients not directly related to a disaster. The increased burden on healthcare services and hospitals during these periods can result in changes in the microbiological flora environment, infection prevention dynamics, and pathogen transmission mechanisms, potentially affecting postsurgical infection processes in patients undergoing nontraumatic surgery. Identifying these effects is crucial for strengthening infection control, optimizing microbiological flora management, guiding the return to normalcy in healthcare services after natural disasters, and directing empirical antibiotic treatments in infectious processes during recovery.

Significant findings were obtained regarding the management of infections during the recovery and postoperative recovery periods following natural disasters. The increasing frequency of polymicrobial and Gram-negative infections and the complexities of their clinical management serve as warning signs. They emphasize the clinical presentations that may develop in infectious processes following the increased healthcare burden caused by natural disasters, such as earthquakes. In such cases, it is crucial to perform more comprehensive culture samples, more carefully interpret culture results showing the growth of a single microorganism and administer broader-spectrum antibiotic treatments than usual until the pathogen profile returns to normal. As our study has shown, this situation concerns not only traumatic but also nontraumatic orthopedic patients. That is, natural disasters indirectly affect both high-risk patients and nontraumatic groups, including those undergoing primary total knee arthroplasty. Therefore, integrating the findings and results observed in our study into infection control methods during natural disasters, which create a burden and instability in healthcare services, may decrease adverse outcomes in patients undergoing arthroplasty.

This study has several limitations. Its retrospective design and relatively small sample size may have limited statistical power, particularly for regression analyses. However, the role of the study center as a tertiary referral center serving a high patient volume, particularly after the 6 February 2023 earthquake, should be considered when interpreting these findings. Additionally, microbial resistance patterns were not evaluated during the microbiological studies, which is another limitation. The study was also unable to systematically document earthquake-related changes in hospital-level operational parameters—such as bed occupancy rates, surgical volumes, staffing levels, or environmental microbiological surveillance—which limits the ability to directly attribute the observed microbiological shifts to specific healthcare system disruptions. Furthermore, as the study included only patients who had already developed PJIs and did not analyze the full denominator of all patients undergoing total knee arthroplasty before and after the earthquake, no conclusions can be drawn regarding the overall incidence or rate of PJIs in either period. These findings should therefore be regarded as preliminary observational evidence requiring validation in larger, multicenter studies.

## 5. Conclusions

Our study shows that patients who developed infections after primary knee arthroplasty in the post-earthquake period presented with more complex clinical cases and a higher proportion of polymicrobial and Gram-negative pathogens. It is noteworthy that these findings emerged despite similar demographic characteristics (age, BMI, comorbid diseases, and smoking) and comparable surgical and laboratory features between the groups. This suggests that earthquakes may alter the microbiological flora of hospital environments independently of patient-related factors, although the retrospective design precludes definitive causal conclusions. Post-earthquake PJIs presented clinically more challenging conditions, including increased intensive care unit admissions, longer hospital stays, repeated surgeries, and multidrug resistance; however, as this study only included patients who had already developed PJIs, no conclusions can be drawn regarding overall PJI incidence or frequency. The results of our study suggest that natural disasters such as earthquakes may indirectly impact nontraumatic orthopedic patients, including those undergoing arthroplasty. These preliminary observational findings indicate that infection control mechanisms and microbiological surveillance should be adapted and implemented to minimize potential negative outcomes in the post-earthquake period.

## Figures and Tables

**Table 1:** Demographic and clinical characteristics of the study.

		Overall (*n* = 56)	Pre-earthquake (*n* = 26)	Post-earthquake (*n* = 30)	*p*-Value
**Age (years)**	Mean ± SD	67.09 ± 4.11	66.81 ± 4.40	67.33 ± 3.91	0.64
**BMI (kg/m^2^)**	Mean ± SD	26.09 ± 3.51	26.42 ± 3.72	25.80 ± 3.36	0.53
**Gender**	Female	33 (58.9%)	15 (57.7%)	18 (60.0%)	0.86
	Male	23 (41.1%)	11 (42.3%)	12 (40.0%)	
**Smoking**	No	46 (82.1%)	21 (80.8%)	25 (83.3%)	0.81
	Yes	10 (17.9%)	5 (19.2%)	5 (16.7%)	
**Diabetes mellitus (DM)**	No	37 (66.1%)	17 (65.4%)	20 (66.7%)	0.92
	Yes	19 (33.9%)	9 (34.6%)	10 (33.3%)	
**Infection timing**	Early (<3 months)	10 (17.9%)	3 (11.5%)	7 (23.3%)	0.41
	Delayed (3–12 months)	11 (19.6%)	4 (15.4%)	7 (23.3%)	
	Late (>12 months)	35 (62.5%)	19 (73.1%)	16 (53.3%)	
**Operation time (min)**	Mean ± SD	-	62.3 ± 17.1	64.4 ± 14.5	0.58
**Hematocrit**	Mean ± SD	-	41.2 ± 9.4	40.1 ± 8.9	0.67
**Albumin**	Mean ± SD	-	3.7 ± 1.0	3.8 ± 1.1	0.74

Grouping was based on the date of microbiological confirmation of PJI (culture positivity), not the date of index arthroplasty.

**Table 2:** Microbiological characteristics and infection patterns before and after the earthquake.

		Pre-Earthquake	Post-Earthquake	*p*-Value
Infectious Agents (Isolate-Based)		(*n* = 32 Isolates)	(*n* = 47 Isolates)	
	MRSA	15 (46.9%)	13 (27.7%)	
	Coagulase-negative Staphylococcus	11 (34.4%)	11 (23.4%)	
	Acinetobacter baumannii	3 (9.4%)	13 (27.7%)	
	Pseudomonas aeruginosa	3 (9.4%)	10 (21.3%)	
	Overall distribution			0.141 ^†^
	Gram-positive	26 (81.3%)	24 (51.1%)	
	Gram-negative	6 (18.8%)	23 (48.9%)	0.021 ^‡^
	Monomicrobial infection	20 (76.9%)	13 (43.3%)	
	Polymicrobial infection	6 (23.1%)	17 (56.7%)	0.011 ^‡^

^†^ Pearson χ^2^ test was used for the overall comparison of infectious agent distribution (4 × 2 contingency table). ^‡^ Fisher’s exact test was used for comparing Gram-positive versus Gram-negative pathogens (2 × 2 table) and for comparing monomicrobial and polymicrobial infection patterns (2 × 2 table). Infectious agent distribution was analyzed on an isolate basis (total isolates = 79), and infection patterns were analyzed on a patient basis (total patients = 56). Gram-positive pathogens: MRSA + coagulase-negative Staphylococcus; Gram-negative pathogens: Acinetobacter baumannii + Pseudomonas aeruginosa. Polymicrobial infection was defined as the isolation of two bacterial pathogens from the same patient.

**Table 3:** Association of diabetes mellitus and smoking with polymicrobial infection.

		Monomicrobial *n* (%)	Polymicrobial *n* (%)	Odds Ratio (95% CI)	*p*-Value
**Diabetes mellitus (DM)**	No	24 (64.9)	13 (35.1)		
	Yes	9 (47.4)	10 (52.6)	2.05	0.257
**Smoking**	No	29 (63.0)	17 (37.0)		
	Yes	4 (40.0)	6 (60.0)	2.56	0.288

Odds ratios were calculated using logistic regression analysis. *p*-values were obtained using Fisher’s exact test. CI: confidence interval. In logistic regression analysis, polymicrobial infections were observed more frequently among patients with diabetes mellitus and smokers. Although these associations were not statistically significant, the odds of developing polymicrobial infection were more than twofold higher in patients with diabetes (OR 2.05, *p* = 0.257) and smokers (OR 2.56, *p* = 0.288).

## Data Availability

The data presented in this study are available on request from the corresponding author.
